# Abdominal Section—Celiotomy, Laparotomy

**Published:** 1901-08

**Authors:** Cecil French

**Affiliations:** Washington, D. C.


THE JOURNAL

OF

COMPARATIVE MEDICINE AND VETERINARY ARCHIVES.

VOL. XXII. *AUGUST, 1901.* No. 8.

### ABDOMINAL SECTION—CELIOTOMY, LAPAROTOMY.

By CECIL FRENCH, D.V.S.,
WASHINGTON, D. C.

CELIOTOMY is a simple and safe operation in the dog. It is exceedingly rare that peritonitis supervenes, even when the precautions amount merely to an observation of the ordinary rules of cleanliness. In fact, it may be unreservedly asserted that the necessity for antisepsis, so far as fear of infecting the peritoneum is concerned, has been greatly overrated. Numerous experiments and abundant clinical observation have demonstrated beyond any doubt that the peritoneum of this animal possesses extraordinary refractory power against the action of pyogenic microörganisms.

The experiments of Wegner[1] and Grawitz[16] have shown that considerable numbers of the ordinary forms of pyogenic microörganisms may be introduced into the peritoneal cavity without any particular effect on the animal, provided the absorptive power of the peritoneum is not impaired. Reichel[17] found that peritonitis developed only when the quantity of putrescible material exceeded that amount, which could be eliminated within a limited time. Waterhouse[18] injected 6 c.c. of cultures of staphylococcus aureus, streptococcus, and intestinal bacteria, respectively, and found the animals survived. He then tried to produce the same conditions which sometimes exist after operations by introducing 8 c.c. of urine and small quantities of blood with the cultures, and still the animals lived. But the presence of considerable quantities of putrescible material, such as blood clots 3 c.c. in size when the cultures were introduced, was followed by death in twenty-four hours. Cats suffering from ascites quickly died from peritonitis, owing to diminished absorptive activity of the peritoneum and the presence of a favorable culture medium. Halsted[19] introduced pieces of sterile potato, and found they became encapsulated without producing any disturbance, but when infected with pyogenic organisms invariably caused peritonitis. Welch[20] made similar observations, and found further that an infected wound readily and uniformly suppurated when it contained masses of tissue strangulated by ligature. He made a large number of experiments by ligating portions of omentum and then injecting cultures of staphylococcus aureus into the peritoneal cavity. In most cases general peritonitis developed, in some cases localized peritonitis, and in others no peritonitis followed the inoculation.

Thus we see that the absorptive power of the healthy peritoneum in the dog is very great, and that there is little risk of peritonitis resulting from introduction of any limited infection from without, such as may occur during the course of an operation when the peritoneum comes in contact with even the unwashed hands. Neither is there any greater risk after it has been sealed by the suturing of the muscular wall. This is due to the well-known fibrinoplastic property of the peritoneum. Wounded peritoneum possesses an extraordinary power of adhesiveness when brought in contact with peritoneum. This fact is beautifully demonstrated during suturing operations of the peritoneum, when fibrinous adhesions may be observed to form and firmly bind apposed surfaces within a period of some half-dozen minutes. The chief danger arises from the presence of much putrescible matter, such as large blood clots and portions of organs isolated from their vascular supply by ligature, etc., and allowed to remain. The slightest infection sustained by quantities of such matter is very liable to lead to general peritonitis. Extravasations from the alimentary tract following imperfect apposition or suturing of surgical wounds of the intestinal walls are always highly dangerous. In the various visceral-suturing experiments that have been carried out on dogs these conditions have been chiefly the cause of fatal termination.

But there is another point to be considered. Good surgery aims to secure healing of all the tissues of a surgical wound by granulation of second intention as distinguished from suppuration, if there cannot always be hope of healing by first intention or primary union. Now, while there is a slight risk of the open peritoneum becoming seriously infected either during the course of or after an operation, it is an entirely different matter when the subcutaneous and cutaneous wounds are dealt with. Yet even with these, healing *per primam* is possible, provided certain favorable conditions exist.

Divided muscle unites very readily by first intention—*i. e.*, by adhesion of the cut edges through organization of inflammatory serum by fibrin, provided the edges are brought into accurate approximation by sutures, and no suppurative process takes place in the subcutaneous connective tissue to hinder.

The part of the wound most difficult to treat is the subcutis. Here the defensive power of the organism is poor, and should there occur any intervening spaces they form suitable pockets for the reception of blood, which readily oozes from the surrounding wounded capillaries and veinlets. Should there be the slightest degree of infection present, the microörganisms, being removed from contact with living tissue, are free to multiply beyond the area of its phagocytic action, and thereby establish abundant suppuration. It is very difficult to suture the subcutis so as to prevent the formation of spaces. Herein lies the chief argument on behalf of asepsis. We must seek to prevent infection of the subcutis. This end is best attained by the previous sterilization by heat of all instruments and absorbent and suturing material. But the employment of chemical disinfectants beyond the outer skin must be avoided. Particularly does this apply to treatment of the peritoneum. Chemical disinfectants tend to irritate and impair the absorptive power rather than to aid it in any way. It is better to rely upon the vital capacity of the living tissue to overcome the destructive tendencies of any pyogenic bacteria that may gain entrance.

With the superficial layers of the skin, however, it is advisable to use some chemical disinfectant after thoroughly cleansing it in the immediate neighborhood of the contemplated incision, but even then absolute disinfection cannot be accomplished. The deep-lying microörganisms cannot be reached by any agent short of a gas. Welch[20] proved that the white staphylococcus occurs in wounds where every possible antiseptic precaution has been taken, but it possesses very feeble pyogenic capacity, and hence seldom exerts interference with the process of healing. Schloffer[21] was able to produce a pronounced abscess in one instance by injecting a pure culture subcutaneously. It is hardly necessary to add that the hands of the operator must be disinfected in the same manner as the skin of the animal.

Because of the remarkable tolerance of abdominal section exhibited by the dog, the practitioner need never shrink from undertaking the operation as an explorative measure. It is not always possible to corroborate a diagnosis of internal lesion by external appearances or palpation. Especially is this the case in plethoric animals and where the lesion is situated in a position remote from the surface of the body. For instance, an animal may exhibit all the symptoms of impermeability of the intestinal canal —intractable vomiting and suppression of defecation, with extreme prostration—and yet the abdominal wall may be so tense as to preclude the possibility of diagnosis by palpation. Again, it is very difficult in gunshot cases to decide whether the intestine or any other organ has been perforated or not. The appearance of the external wounds has no diagnostic value, since there is no gaping of the parts owing to contraction of the abdominal muscles, and it is often impossible, and in most cases inadvisable, to use a probe. Sometimes estruation continues after ablation of the ovaries. This is generally due to the persistence *in situ* of a portion of ovarial tissue, which can be ascertained by an explorative operation. It is recognized that the sudden accidental application of a violent compressing force to the abdomen when the bladder is distended is very apt to cause rupture of the latter or even of other organs and bloodvessels. In such cases it is a wise procedure to open the cavity when there is evidence of systemic collapse. Internal hemorrhage through rupture of even lesser vascular branches is always dangerous. Divided vessels of the abdominal cavity possess a remarkable tendency to bleed persistently. If, however, air be admitted through abdominal section the conditions are quickly altered, clots commencing to form. Such vessels should, however, always be secured to guard against a recurrence of the hemorrhage when the cavity is closed and it thereby returns to its former condition.

The operation should invariably be performed with the subject under the influence of an anæsthetic. Not only do humane considerations demand this, but the accurate conduct of a delicate operation on a struggling animal is an impossibility. Before the abdomen is opened every possible contingency must be fully considered, so that the necessary instruments, surgical aids, and sutures be prepared, rendered aseptic, and laid handy.

If it be possible to arrange, the animal should receive no food for twenty-four to forty-eight hours previously, and also receive a purgative. A distended bowel is always a particular annoyance to the operator by reason of its tendency to extrude itself.

With regard to the selection of a site for section, it may be said there are two main positions—the lateral and the median. Each has its advocates, and without doubt each certain advantages over the other. But it must be borne in mind that no absolute rule can be laid down in the matter. Neither position is peculiarly suitable for reaching every organ, and the operator must be governed by the conditions present. Most of the organs can be reached by the median line, and this position has much to commend it. It can be performed almost bloodlessly, it can be easily enlarged, it affords better access to all parts of the cavity for explorative purposes, and it permits of perfect drainage. Further, any resultant scar is not observable when the animal is in the standing position. The chief objection offered against it is said to be the greater risk of the dissected parts failing to become united. I cannot concur in this opinion, never having experienced the misfortune of hernia. La Torre[2] holds that such risk is reduced to a minimum if the incision be made through the muscular tissue of the rectus abdominis, slightly to one side, and not through the aponeurotic tissue of the linea alba. Union of muscular fibre, particularly by first intention, is always stronger than union by granulating cicatricial tissue. Human surgeons recognize that the commonest factor in the development of hernia is an infection causing the wound to fill in slowly with scar tissue. Median section has a disadvantage in males, in that the wound may become soaked with urine. Even if the incision be made posterior to the preputial orifice, and this difficulty thereby avoided, there still remains a pronounced tendency to the development of suppurative processes. The reason for this is to be attributed to the proximity of the penial mucosa, which is so often the seat of catarrhal discharges, and whence microörganisms can so easily be transmitted to the wound during the course of an operation.

In the lateral position the risk of hernia resulting is almost *nil*.

Among the drawbacks are: the greater thickness of muscular tissue which must be divided, the necessity of securing the epigastric vessels, and the tendency of pus to burrow between skin and wall, and even into the peritoneal cavity in the event of the wound suppurating during healing. Should purulent peritonitis intervene, either from such burrowing or incident to secondary operations on internal organs, the chances of recovery are remote, in consequence of absence of drainage.

Generally speaking, the organs are best reached as follows: the stomach, spleen, pancreas, and liver, in the anterior third—*i. e.*, immediately posterior to the thorax—the ovaries and intestines exactly in the centre of the distance between the ensiform process and the symphysis pubis; the uterus, bladder, ureters, and rectum immediately anterior to the pubis.

When the operation is undertaken as an explorative measure the surgeon is, figuratively speaking, groping in the dark. In such instances the middle third should be chosen.

Instances have been recorded where it has been found necessary to close the first incision and make a second one before the seat of lesion could be reached. Venneholm[3] described an operation for fecal impaction, the mass of which was lying in front of the pubic bone. This mass was mobile, and the operator expected to reach it without any trouble. The first incision was made in the linea alba, but the obstructed portion of the bowel could not be extracted. It was then necessary to make a second incision to the side of the prepuce.

Gluck,[4] in his experimental extirpation of the liver, found the organ could be reached most conveniently by incising from the ensiform process to the costo-vertebral articulation of the eighth rib, and resecting the eighth and ninth ribs. Griffiths,[5] in his experimental surgery of the pelvic viscera, found he could expose the latter to better advantage by dividing the symphysis pubis and then separating both sacro-iliac synchondroses by forcibly turning the iliac bones outward. The bones can be separated two inches or more.

The animal being secured in the proper position with hopples, the skin in the immediate vicinity of the contemplated incision is clipped or shaved of its hair and scrubbed with warm water and soap and some disinfectant solution. The incision is made with a sharp scalpel and should not be less than two inches in length in the smallest animals, while in the larger breeds it may be found necessary to make the wound large enough to admit the whole hand. To reach the cæcum and kidney always requires a large incision, owing to their remote position. The skin, subcutaneous connective tissue, and muscles are successively divided, the fibres of the latter being separated according to the direction in which their course runs. Three muscular coats require to be divided in the extreme lateral position—the obliquus externus, the obliquus internus, and the transversalis. In the median line there are the aponeuroses of these muscles and a single true muscular coat—the rectus. In the male prepubic median section is made by incising the skin immediately to one side of the penis and dislocating the latter —*i. e.*, by pushing it in the opposite direction. In making this incision one must avoid wounding the posterior epigastric vein—a prominent vessel which runs on either side a short distance from the penis. There is always slight hemorrhage in this region. Section of the muscular wall can then be made in the median line as in females. Froehner[6] believes he can guard against contact with urine and secure better prospect of healing *per primam* in males by making always a lateral incision about one and one-half to two inches to one side of the linea alba, and subsequently painting the surface of the wound with a 20 per cent. solution of iodoform in ether. Stoss[7] opens the muscular wall by thrusting a grooved director through at one commissure of the skin incision, after making the latter, and passing it with the groove uppermost in contact with the inner surface of the wall along a line corresponding to the contemplated incision. There is no danger of piercing the bowel with a blunt director, and if any portion of the former should be caught up it is perceptible through the wall as a slight elevation. In that case the director is withdrawn far enough to release the gut and again passed. With the director as a guide, the incision in the muscular wall is made with a bistoury.

Any vessel being divided, it is grasped with hæmostatic forceps, which generally suffices to arrest the flow within a minute or two. The epigastric vessels should always be ligated. All hemorrhage being under control, the peritoneal coat may be picked up with the dissecting forceps and pierced with the scalpel, or it may be gently incised *in situ*, and the opening enlarged with the finger. Beneath is found the omentum major, excepting just in front of the pubic border. It may be gently pulled away from the hypogastric region and stowed away in the epigastric, or an opening may be made in it by tearing at a point opposite the incision.

The viscera are now exposed to view, and the necessary supplemental operations demanded by the exigencies of each particular case are immediately undertaken.

There is generally some tendency to protrusion of intestinal coils. This must be guarded against as much as possible, though it is rare that any evil effects follow prolonged exposure. It may be prevented by temporarily inserting flat sponges or small cloths (sterilized) just within the wound. The radiation of the heat incident to prolonged exposure tends to lower the vitality of the peritoneum, whereby its eliminative or absorptive power is checked. Vincent[8] in his experiments found that there was more likelihood of peritonitis developing after exposure of the bowel, and regarded it as important not to let any escape. Should it be necessary to allow of any considerable protrusion of viscera it is advisable to carefully protect the exposed organs with sterile gauze wrung out in hot water and repeatedly applied. It is a good plan, when an operation is likely to last a considerable time, to employ a "celiotomy cloth." This consists of a piece of cloth with a slit in it made to correspond with the skin incision, and sterilized. It is laid over the abdomen, and thus prevents contact of protruding organs with the skin. A full bladder, which is often an interference, may be emptied by direct pressure.

The pelvic cavity is opened by extending the skin incision to the hinder border of the symphysis pubis (passing to one side of the penis in the male). The symphysis is cut by means of a strong knife or small hand-saw. One must avoid injuring the dorsal veins of the penis in the male and the plexus of the veins from the clitoris in the female, as hemorrhage therefrom is somewhat difficult to control. A small block of wood is placed under the sacrum, and the iliac bones forcibly turned outward so as to produce a fracture-dislocation at the sacro-iliac synchondrosis. Restitution of continuity of these parts is accomplished by wiring the bones at the symphysis according to the methods employed in bone suturing.

When it is desired to close the abdominal wall a careful inspection must be made to ascertain whether any blood clots or other putrescible material or sponges remain in the cavity. These are to be removed, as their presence is conducive to peritonitis.

If the omentum has been misplaced it should be returned as nearly as possible to its original position. Any rents in this organ should be sutured, otherwise there is risk of a loop of bowel passing through the same, when the conditions would be ripe for strangulation. Though I have never known strangulation to result from such conditions, once, while performing a necropsy, I found a coil of small intestine protruding through a rent I had made some two weeks previously in the course of a resection experiment.

In intestinal operations the omentum is sometimes soiled, in which case it may be advisable to remove the contaminated portion, but it is very important to securely ligate any bleeding vessels. In one of Senn's[9] experimental cases it was deemed advisable to remove a portion of the omentum. The animal died the next day owing to hemorrhage of the omentum by slipping or loosening of a catgut ligature. Senn advises against ligaturing of the omentum or mesentery *en masse*, but each individual vessel should be searched for and secured separately with aseptic silk. One reason for this is that tissues often shrink after operation, whereby ligatures become loosened, so that it is dangerous to include a large area in a single ligature. Parkes[10] has also pointed out that the stumps of ligated omentum tend to give rise to trouble through mortification of the occluded end. But, unless the conditions actually demand its removal, it is bad surgery to excise this organ. In certain cases of hernia where its reduction would present considerable difficulty it may be removed with advantage. The omentum performs an important function in the healing of abdominal and visceral wounds. It plays the part of an operculum, invariably becoming adherent to the internal face of the wound or to wounded surfaces of organs.

Because of this protective capacity of the omentum, which is in reality a fold of peritoneum, it is quite unnecessary to stitch the parietal peritoneum.

In bringing the edges of the muscular wound into contiguity some operators apply independent sets of sutures to each of the divided coats. Others use but one set of sutures to include all the coats. I have found the continuous suture best adapted to the purpose. In the median position there is but one small muscular coat to unite, though the aponeuroses of the others should be included. Much of the strength of the abdominal wall lies in the fascia in front of the recti muscles. When interrupted sutures are used no stitch should be tied until all are inserted, the curved needle being employed, and then tying is to be commenced at each commissure and gradually completed toward the centre. When the opening has been made directly through the linea alba, La Torre advises that the aponeurotic tissue be removed as far as the muscular substance of the recti muscles, owing to the yielding tendency displayed by cicatrices of the former class of tissue.

When the epigastric artery and veins have been tied, the ligatures are very apt to become displaced or slip while the sutures are being applied to the wall. This accident may escape the operator's notice, and a fatal hemorrhage result. Znamensky[11] lost a case in this manner. Wherefore, careful attention should be paid to this matter. The best suturing material for the muscular wall is silver wire, which may be allowed to remain permanently in position.

It must be borne in mind that our best efforts should be put forth to obtain healing of the muscular wall by first intention—*i. e.*, by adhesion of the cut edges of the muscle through organization of inflammatory serum by fibrin. So long as we can secure rapid reunion of the divided muscle with a minimum formation of connective tissue the strength of the wall is little impaired, and the chances of a resultant hernia are remote.

The importance of securing accurate approximation of all divided subcutaneous tissue cannot be overestimated. The formation of spaces must be guarded against as much as possible, for, as has already been pointed out, such spaces, if infected, form suitable foci for suppuration. The reason why pus is so apt to form in males is owing to the proximity of the penial mucosa, which is so commonly the seat of catarrhal disorder, and the ease with which bacteria are carried thence by the tongue of the animal or by the surgeon during the course of an operation. The wound made when the penis is dislocated in order to reach the median line is particularly prone to suppurate. The connective tissue in this locality is deep, and when divided tends to form quite a cavity under the sutured skin. Therefore, it is always a wise precaution to draw the divided subcutis together with a few silver-wire sutures whenever any gaping is evident. For the skin by far the best suture is the subcuticular, insuring, as it does, the utmost protection from infection from without. Any of the non-absorbable material may be used, as it is easily removed.

The wound should be examined closely for the succeeding day or two for signs of suppuration, and if such be discovered it must be promptly opened and the matter evacuated. A subcutaneous abscess without drainage is always dangerous. Indeed, fatal terminations have been recorded where such seemed to have been the sole cause of death either through septicæmia or pyæmia. Peterson[12] lost two cases in this manner, eight and thirteen days after the operation, respectively. Froehner says they are productive of septic endocarditis. Where there is no infectious disease of the teeth, or no discharging wound or diseased process present, whereby infection of highly virulent microörganisms could be transmitted by the tongue of the animal, anything in the nature of a protective bandage is best dispensed with, particularly when the subcuticular suture is employed. As a rule, a dog soon learns to work its muzzle in under a bandage to lick the wound. But, in the excepted instances noted, it is advisable to protect the wound as much as possible with gauze and linen bandages and a plentiful supply of disinfectant powder.

An animal that has been subjected to laparotomy should be restrained from taking active exercise for a few days, so that no risk be run of the sutures tearing out from some sudden movement.

Occasionally, non-absorbable sutures in the muscular wall fail to become encapsulated, and a fistulous tract is established long after apparent healing of the skin has taken place. In such cases a director must be passed into the extremity of the sac, and by means of a curved bistoury sufficient of the parts laid open again to permit of the offending thread being extracted.

As has already been stated, purulent peritonitis occurring as a result of intestinal perforation owing to imperfect suturing in secondary operations, or from the presence of putrescible material, or other causes, is an occasional sequel. If from symptoms of collapse or local manifestations such a condition can be diagnosed, no time must be lost in reopening the cavity to establish drainage, either in the same position or a new one. Internal lesions must be attended to and the cavity irrigated with moderately hot water.

Kummer[15] related an instance of a dog tearing out the abdominal sutures three weeks after operation, and succumbing as a result thereof in thirty-six hours, and Moeller[14] recorded a similar occurrence. Caution must be observed in the feeding of an animal subsequent to this operation. Hobday[15] found that a hearty meal of solids is apt to induce violent peristalsis after the bowel has been at rest for a longer or shorter period, and may cause tearing out of the sutures and protrusion of the intestine through the abdominal wound.

### REFERENCES.

1. Verhandl. d. Deutsch Gesell. f. Chir., Berlin, 1877.
2. La Gynéc., April, 1897.
3. Thieraerztliches Centralblatt, June 10, 1898.
4. Langenbeck's Archiv. f. Chirurg., vol. xxviii. p. 3.
5. Journal of Anatomy and Physiology, 1894–95, vol. xxix. p. 62.
6. Monatsheft. f. prakt. Theirheilk., 1893–94.
7. Ibid., 1896–97.
8. Rev. de Chir., 1881, p. 556.
9. Intestinal Surgery, p. 181.
10. Gunshot Wounds of the Small Intestines, p. 27.
11. Langenbeck's Archiv f. Chirurg., vol. xxxi. p. 149.
12. Journal of the American Medical Association, 1901, p. 808.
13. Langenbeck's Archiv f. Chirurg , vol. xlii. p. 534.
14. Lehrb. d. spec. Chir. f. Thieraerzte.
15. Journal of Comparative Pathology and Therapeutics, September, 1899.
16. Char. Annal. Jahr., 1886, p. 9.
17. Deutsch Zeitschr. f. Chirurg., 1889, p. 30.
18. Virchow's Archiv, 1890, vol. cxix. p. 342.
19. Johns Hopkins Hospital Reports, 1891, p. 2.
20. Transactions of the Congress of American Physicians and Surgeons, 1891, vol. ii. p. 1.
21. Langenbeck's Archiv f. Chirurg., 1898, vol. lvii. p. 334.

